# Wake-Up Stroke: Clinical Characteristics, Imaging Findings, and Treatment Option – an Update

**DOI:** 10.3389/fneur.2014.00035

**Published:** 2014-03-26

**Authors:** D. Leander Rimmele, Götz Thomalla

**Affiliations:** ^1^Klinik und Poliklinik für Neurologie, Kopf- und Neurozentrum, Universitätsklinikum Hamburg-Eppendorf, Hamburg, Germany

**Keywords:** wake-up stroke, acute ischemic stroke, thrombolysis, computed tomography, magnetic resonance imaging, fluid attenuated reversion recovery, DWI-FLAIR-mismatch

## Abstract

About 25% of all strokes occur during sleep, i.e., without knowledge of exact time of symptom onset. According to licensing criteria, this large group of patients is excluded from treatment with received tissue-plasminogen activator, the only specific stroke treatment proven effective in large randomized trials. This paper reviews clinical and imaging characteristics of wake-up stroke and gives an update on treatment options for these patients. From clinical and imaging studies, there is evidence suggesting that many wake-up strokes occur close to awakening and thus, patients might be within the approved time-window of thrombolysis when presenting to the emergency department. Several imaging approaches are suggested to identify wake-up stroke patients likely to benefit from thrombolysis, including non-contrast CT, CT-perfusion, penumbral MRI, and the recent concept of diffusion weighted imaging-fluid attenuated inversion recovery (DWI-FLAIR). A number of small case series and observational studies report results of thrombolysis in wake-up stroke, and no safety concerns have occurred, while conclusions on efficacy cannot be drawn from these studies. To this end, there are ongoing clinical trials enrolling wake-up stroke patients based on imaging findings, i.e., the DWI-FLAIR-mismatch (WAKE-UP) or penumbral imaging (EXTEND). The results of these trials will provide evidence to guide thrombolysis in wake-up stroke and thus, expand treatment options for this large group of stroke patients.

## Methods

The literature used in this review was searched in PUBMED and MEDLINE (1977–2013 October) using the following key words “wake-up stroke,” “stroke on awakening,” “stroke of unknown symptom onset,” “thrombolysis,” “diffusion weighted imaging,” and “fluid attenuated inversion recovery (FLAIR).” The selection was made by the authors evaluating clinical relevance, currentness, and methodical correctness. Our purpose was not to give a general overview of the current state of literature concerning thrombolysis in wake-up stroke in general, but to emphasize the possibilities of treatment for wake-up stroke on the basis of current literature. Therefore, we have done no systematic literature review but one focused on our demand.

## Stroke and Stroke Thrombolysis

Patients waking-up with symptoms of stroke represent a specific subgroup of stroke patients. In general, stroke is the second most common single cause of death and the most frequent cause of permanent disability in industrialized countries. Based on WHO estimates about 15 million people suffer from stroke each year of whom five million are left permanently disabled (1). As a consequence, stroke carries an enormous social and economic burden both for the individual patients as for society at large. In the EU stroke accounts for just over 500,000 deaths each year with just around 1 in 10 men (9%) and 1 in 8 women (12%) dying from stroke (2).

The demonstration of efficacy and safety of treatment with intravenous tissue plasminogen activator (IV-tPA) (3) has ended an age of therapeutic nihilism and revolutionized stroke care. Together with the establishment of the stroke-unit concept, thrombolysis has motivated the implementation of specialized acute stroke treatment in most developed countries.

In ischemic stroke, the acute occlusion of a brain vessel leads to hypoperfusion of the downstream brain areas resulting in an insufficient supply with oxygen. Within seconds the functional metabolism of brain cells breaks down, and depending on the degree and duration of ischemia, the affected brain areas decay. Thrombolysis aims to reperfuse the brain vessel occlusion by dissolution of the clot. In acute stroke, thrombolysis was proven effective in two randomized controlled trials resulting in a marked increase of the number of patients with a favorable outcome after stroke (3, 4). In large observational trials, this effect was reproduced in the clinical practice (5), and thrombolysis has become a keystone of acute stroke treatment. However, thrombolysis was only proven effective in patients treated within 4.5 h of symptom onset, and is only recommended for patients to be treated within this time-window.

## Wake-Up Stroke

In a large number of stroke patients, the time point of symptom onset is not known. About 20–25% of stroke patients realize stroke symptoms after waking-up from sleep (6–9) (Table 1). This subgroup of stroke patients (“wake-up strokes”) differs from stroke patients who suffer from stroke while being awake and pose a specific challenge to stroke physicians. The most relevant difference between both groups is the fact that in wake-up stroke patients, the exact time point of symptom onset is unknown. As a result, according to approval criteria and guideline recommendations, this large group of patients is excluded from thrombolysis (9, 10), thus excluding patients from the only approved specific treatment of acute stroke with proven safety and efficacy.

**Table 1:** **Incidence of wake-up stroke**.

Wake-up strokes (%)	Total	Reference
100 (27)	364	(11)
301 (24)	1.248	(8)
349 (14)	2.585	(12)
48 (18)	263	(13)
273 (14)^a^ (+12% “no estimation of onset”)	1.854	(6)
5.152 (30)	17.398	(14)

*^a^Of note, according to clinical in- and exclusion criteria 36% of these patients were potentially eligible for thrombolysis according to the authors*.

Within the past years, however, wake-up stroke has come into focus of research activities. Observational studies have brought insights into clinical and imaging characteristics of wake-up stroke; new approaches to guide treatment in wake-up stroke patients have been suggested. Finally, clinical trials are underway testing intravenous thrombolysis in patients with unknown time of symptom onset including wake-up stroke. We will give an update of the recent insights on wake-up stroke and ongoing developments that are likely to improve the treatment of these patients in the near future.

## Clinical and Imaging Characteristics of Wake-Up Stroke

There are observations that point toward strokes during sleep being more severe (15) and having a worse clinical outcome (12). In a recent large analysis of wake-up-stroke as compared to stroke while awake, a smaller initial severity for wake-up-stroke with deterioration to comparable mortality and morbidity was shown (14). Especially, the secondary deterioration underlines the potential responsiveness of wake-up stroke for therapy. In addition, further clinical and imaging observations suggest that in a large number of patients waking-up with stroke symptoms strokes may have occurred in the early morning hours so that they might still be eligible for thrombolysis. There are studies reporting comparable frequency of early ischemic signs on CT (EICs) in wake-up stroke patients as compared to patients studied by CT within 3 (16) or 6 h of symptom recognition (17). Additionally, the equal developmental time pattern of EICs in patients with known and unknown onset in a follow-up from 3 h after recognizing the symptoms to 3 months supports the presumption that wake-up strokes occur close to awakening (18). An MRI-based study reported a similar proportion of wake-up stroke patients showing a perfusion–diffusion-mismatch as compared to patients within 3 h of symptom onset (11). A similar observation was made for the detection of “tissue at risk” by perfusion CT in wake-up stroke patients (18). Together these findings suggest that a large number of patients with wake-up stroke might still be within a time-window for thrombolysis when reaching the hospital.

## Imaging Approaches to Guide Treatment in Wake-Up Stroke

**Figure 1:**
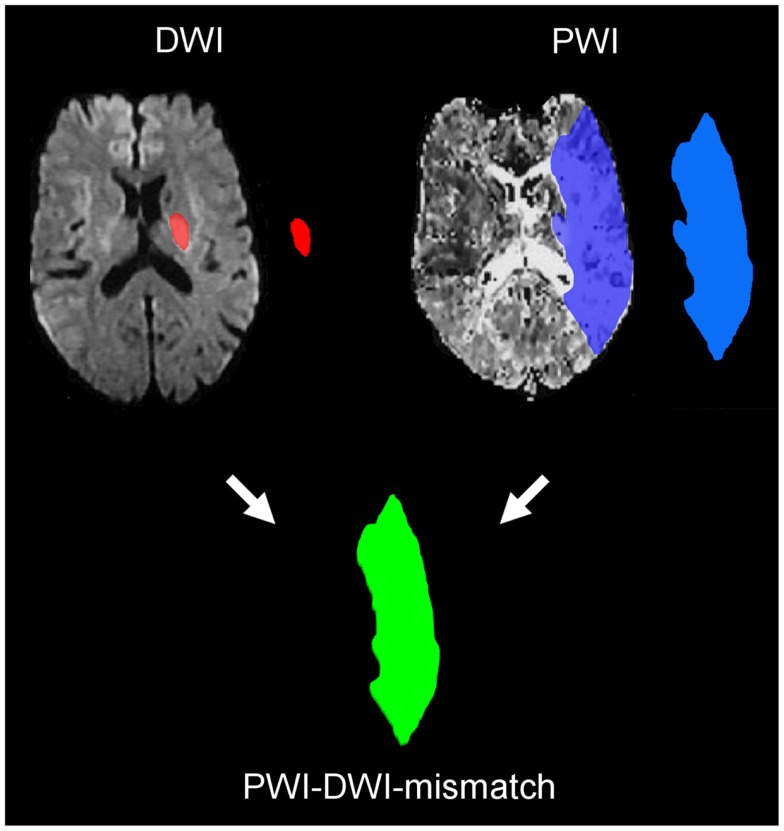
**MRI perfusion–diffusion mismatch**. The small lesion on diffusion weighted imaging (DWI) represents the infarct core, while the much larger area in the time to peak map calculated from perfusion imaging (PWI) identifies the area of critically hypoperfused tissued. The mismatch between both volumes represents the tissue at risk of infraction and thus, the target tissue for reperfusion treatment.

To identify wake-up stroke patients who may benefit from thrombolysis two imaging approaches are set in the focus of investigation. The detection of tissue at risk by penumbral imaging (perfusion–diffusion mismatch, Figure 1), and the approach of identifying stroke patients within the 4.5 h-time-window by tissue characteristics in stroke MRI [diffusion weighted imaging (DWI)-FLAIR-mismatch]. Penumbral imaging based on DWI and perfusion MRI allows to determine the status of damaged tissue in acute ischemia and to distinguish irreversibly damaged one from critically hypoperfused but potentially salvageable tissue. The mismatch of these was suggested to identify tissue that is tPA-responsive beyond time-window or independent from time (19–21). In observational studies, it was shown that tissue at risk may be saved by reperfusion up to 6 h after symptom onset (22). Secondary analysis of randomized controlled trials with selection by penumbral imaging (23, 24) demonstrated a reduction of final infarct volume (25) and a clinical benefit of thrombolysis in patients with a large perfusion–diffusion mismatch with an odds ratio for good clinical response of 2.83 between desmoteplase and placebo (26). Moreover, large DWI lesion volumes were found to be associated with a higher risk of symptomatic intracranial hemorrhage (SICH) and poor outcome (22, 27, 28). Thus, the exclusion of patients with very large DWI lesion volumes by MRI will likely increase the safety of MRI-based thrombolysis. The re-analysis of EPITHET and DEFUSE resulted in a more restrictive definition of the perfusion lesion (29, 30), which are used in the ongoing Extending the time for Thrombolysis in Emergency Neurological Deficits (EXTEND) trial (31). The detection of treatable wake-up strokes by perfusion–diffusion mismatch has been suggested (32–34) and used in a relatively small non-randomized trial (35). In line with these considerations, the EXTEND trial will allow the randomization of patients with wake-up stroke based penumbral imaging.

**Figure 2:**
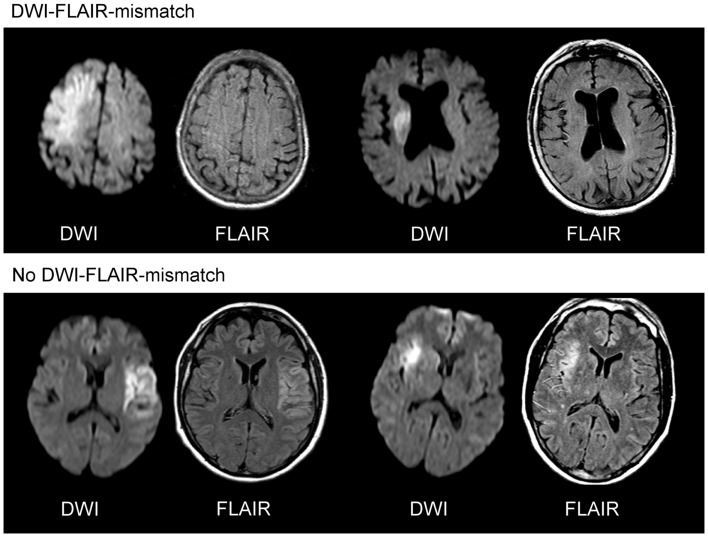
**DWI-FLAIR-mismatch**. The upper row gives two examples of a clearly visible acute ischemic lesion on diffusion weighted imaging (DWI), while no marked parenchymal hyperintensity is detected on fluid attenuated inversion recovery (FLAIR) images indicating DWI-FLAIR-mismatch. In the lower row, a clear hyperintensity can be seen on FLAIR images in the area of the acute DWI lesion (no DWI-FLAIR-mismatch).

The DWI-FLAIR-mismatch (Figure 2) concept refers to the time-window. There is a broad and striking evidence for the benefit of intravenous thrombolysis of ischemic stroke within 4.5 h (3, 4, 35). Therefore, it was suggested to use brain imaging to determine stroke age in the case of unknown symptom onset. The chronological evolution of ischemic stroke can be characterized by MRI. A lack of cerebral blood flow with a decreased intracellular energy metabolism causes cytotoxic edema, which can be detected by a reduced apparent diffusion coefficient (ADC) on DWI within minutes of stroke (36, 37). During the following 1–4 h, tissue osmolality increases, accompanied by a net increase of water (38, 39). This absolute increase of water content can be detected by T2-weighted MRI (36, 40). Thus, DWI allows an instant determination of acute ischemic lesions, but gives no evidence of further developmental changes, which may be characterized by T2. Due to artificial limitations of T2 caused by the high signal intensity of cerebrospinal fluid (CSF) with partial volume effects FLAIR is considered superior and more widely used (41, 42). The pattern of a visible ischemic lesion on DWI together with normal T2-weighted imaging or FLAIR is a typical finding in human stroke if imaging is performed within the first hours of stroke (43–45). These results are also well in line with data from experimental stroke, where T2wI failed to detect acute ischemia until about 2–3 h of stroke (37, 46, 47).

The DWI/FLAIR-mismatch was established (48) to estimate lesion age and identify patients likely to benefit from thrombolysis. It differs from the perfusion–diffusion coefficient by revealing information about the time and not the quality of damage. Single-center studies reported of a 100% visibility of ischemic lesions after 3–6 h (49–51), and the DWI-FLAIR-mismatch was shown to identify patients within 3–4.5 h with high specificity and positive predicted value (PPV) (48–51). These results have been confirmed in large multicenter studies (PRE-FLAIR: PREdictive value of FLAIR and DWI for the identification of acute ischemic stroke patients ≤3 and ≤4.5 h of symptom onset – a multicenter study including 643 patients) (52). The specificity of the DWI-FLAIR-mismatch to identify patients within the time frame of 4.5 h was in this study 0.81 and the PPV 0.87. The identification within 6 h showed a PPV of 0.95 in PRE-FLAIR (50), and 1 of 0.97 in a Japanese study (49). Concerning the Cochrane analysis indicating a possible beneficial effect of thrombolysis in addition with the absence of an increased risk of SICH up to 6 h of symptom onset (53, 54), these results are of paramount interest for treatment indication. Just recently, an observational study reported of a high frequency (44%) of wake-up strokes showing a DWI-FLAIR-mismatch (55). Based on this evidence, the *Efficacy and safety of MRI-based thrombolysis in wake-up stroke: a randomized, double-blind, placebo-controlled trial (WAKE-UP)* will randomize wake-up stroke patients using the DWI-FLAIR-mismatch as the imaging criterion to identify patients likely to benefit from thrombolysis (56).

## Thrombolysis in Wake-Up Stroke: Case Reports and Observational Studies

Resulting from the dissatisfaction with the lack of any evidence-based treatment recommendations for patients with wake-up stroke, there is a growing number of case reports and case series, which report on thrombolysis in patients with wake-up stroke based on imaging findings (Table 2). These studies used either plain CT (50), multiparameteric CT (57–60), or multiparametric stroke MRI (32–35). In a study including 74 patients with an onset time over 4.5 h and 73 patients with an unknown onset no difference in eligibility and response for perfusion CT based thrombolysis was shown (59). Non-contrast CT and clinically indicated thrombolysis for wake-up ischemic stroke in over 80 years old patients was considered to be beneficial concerning the modified Ranking Scale (mRS) after 90 days compared to non-thrombolysed patients (61), and a comparison with initial and follow-up examination (mRS) after 90 days of 68 patients with wake-up ischemic stroke to 326 patients within the time-window of 4.5 h showed equal results after thrombolysis indicated by non-contrast CT scan combined with clinical judgment (62). In a further single-center observational safety study 20 patients were treated with intravenous thrombolysis in the presence of an arterial occlusion on CT-A and an ASPECTS score of greater than five on baseline CT (60).

**Table 2:** **Trials with thrombolysis with indication set by imaging in wake-up strokes**.

Sample size wake-up stroke	Imaging method	Main results	Reference
68 Strokes with unknown onset, all received IV-tPA (case–control comparison)	NECT	Similar outcome as treatment within 4.5 h (mRS after 3 months, any ICH, symptomatic ICH)	(61, 62)
73 Strokes with unknown onset, in 32 (44%) of these IV-tPA	Perfusion CT	No SICH, 56% good outcome after 3 months (mRS <2)	(59)
89 Strokes with unknown onset, in 20 (22%) of these thrombolysis	NECT and CT-A/TCD	Two asymptomatic ICH, none symptomatic, two died of massive infarction, two died of stroke complications	(60)
80 Wake-up strokes, 46 received thrombolysis (intra-arterial, IV-tPA, or combined)	NCCT, CT- or MRI-perfusion–diffusion mismatch	Two symptomatic ICH, better clinical outcome (mRS) and higher mortality in treated cohort	(57)
43 Strokes with unknown onset, 10 (22%) received IV-tPA	Perfusion–diffusion mismatch (MRI)	One asymptomatic ICH, no symptomatic ICH	(32)
32 Strokes with unclear onset	Perfusion–diffusion mismatch and FLAIR(non-quantitative)	No difference in frequency of symptomatic ICH and 3 months outcome (mRS after 3 months) to treatment within 4.5 h	(33)
430 Strokes with unknown onset, in 83 (19.3%) of these thrombolysis (10% IV-tPA only)	Perfusion–diffusion mismatch and FLAIR(non-quantitative)	Benefit of treatment (after 3 months: 44.6% mRS 0–2; 28.9% mRS 0–1) with safe MRI-based indication (symptomatic ICH in 6%)	(35)

*IV-tPA, intravenous tissue plasminogen activator; NECT, non-enhanced computed tomography; CT-A, CT-angiography; TCD, transcranial Doppler ultrasound; FLAIR, fluid attenuated reversion recovery; ICH, intracranial hemorrhage*.

RESTORE (reperfusion therapy in unclear-onset stroke based on MRI evaluation) was an observational study in which 83 of 430 patients with an unclear onset of symptoms received tissue-plasminogen activator (rt-PA), intravenously, intra-arterial, or in combination. The decision for applying this therapy was made upon a perfusion–diffusion mismatch of more than 20% and negative or subtle hyperintensities on FLAIR. The clinical outcome determined by the mRS after 3 months was in 44.6% favorable and in 28.9% excellent compared to untreated patients (35). Intracranial hemorrhage with a neurological decline was reported in 6% of the treated patents. Therefore, a distinct benefit for patients being treated with rt-PA due to estimation by MRI could be shown. Restraints of this study are the non-randomized study design, the non-quantitative measurement of FLAIR, and the relatively small number of treated patients with unknown symptom onset.

Finally, there is a single-armed observational US American study of thrombolysis with Alteplase in patients with unknown symptom onset (MR WITNESS: a Study of Intravenous Thrombolysis with Alteplase in MRI-Selected Patients, ClinicalTrials.gov. Identifier: NCT01282242). MR WITNESS will use the concept of DWI-FLAIR-mismatch to identify patients likely to respond to thrombolysis and plans to enroll 80 patients.

In summary, these studies demonstrate the feasibility of imaging guided thrombolysis in wake-up stroke patients while there was no excess in SICH and outcome appeared in large parts similar as compared to thrombolysis in patients treated within 4.5 h of known symptom onset. However, final conclusions to the safety and efficacy of thrombolysis in wake-up stroke can only be drawn from randomized clinical trials.

## Randomized Controlled Clinical Trials of Thrombolysis in Wake-Up Stroke

Currently, two randomized controlled trials of intravenous thrombolysis allow the enrollment of patients with wake-up stroke: WAKE-UP and EXTEND.

EXTEND (ClinicalTrials.gov. Identifier: NCT00887328) is a randomized, multicentre, double-blinded, placebo-controlled phase III trial of intravenous thrombolysis with rt-PA in ischemic stroke patients (31). Treatment has to be initiated between 3 h (or 4.5 h depending on local practice) up to 9 h of symptom onset, or in case of wake-up stroke. For wake-up stroke, the midpoint between sleep onset (or last known to be normal) and time of waking-up must not exceed 9 h. Further clinical inclusion criteria include a National Institutes of Health Stroke Scale (NIHSS) score of 4–26. Patients are studied by MRI including diffusion and perfusion MRI or CT including CT-perfusion and randomized to either treatment with placebo or Alteplase (0.6 or 0.9 mg/kg bodyweight based on local practice) if they show a penumbral pattern on MRI or CT. Penumbral pattern is defined by infarct core volume <70 ml, perfusion lesion/infarct core mismatch ratio >1.2, and absolute mismatch >10 ml. For definition of the perfusion lesion Tmax >6 s for MRI and CT is used, while infarct core is defined using MRI diffusion imaging or CT-CBF imaging. The primary outcome measure is a favorable outcome defined by a score of 0–1 on the mRS at day 90. EXTEND aims to enroll 400 patients in Australia and in an accompanying study in international study sites (EXTEND international). There is a European companion study under preparation (ECASS 4-EXTEND-Europe), which will only use MRI for patient enrollment.

WAKE-UP (ClinicalTrials.gov. Identifier: NCT01525290) is the first clinical trial to use the novel approach of DWI-FLAIR-mismatch to prospectively identify patients for thrombolysis. WAKE-UP is an investigator-initiated, interventional, randomized, double-blind, placebo-controlled, parallel-assignment, international, multi-center efficacy, and safety study (56). The aim of WAKE-UP is to test efficacy and safety of MRI-based intravenous thrombolysis with rt-PA (Alteplase) in patients with unknown symptom onset, e.g., patients waking-up with stroke symptoms who otherwise fulfill the approval criteria for intravenous thrombolysis in acute stroke. Patients fulfilling clinical inclusion and exclusion criteria will undergo MRI including DWI and FLAIR. They will be randomized 1:1 to either treatment or placebo if MRI is indicative of lesion age of less than 4.5 h, i.e., shows a DWI-FLAIR-mismatch. Clinical inclusion criteria include age between 18 and 80 years and a disabling neurological deficit. Primary efficacy endpoint is favorable outcome defined by a score of 0–1 on the mRS 90 days after stroke. Primary safety endpoints are Mortality and death or dependency 90 days after stroke. WAKE-UP plans to enroll 800 patients in 40–60 study sites in six European countries and has started recruitment in October 2012.

## Conclusion

Patients waking-up with stroke symptoms represent a large group of stroke patients who are currently excluded from intravenous thrombolysis based on licensing criteria. Growing evidence from clinical and imaging studies suggests that a relevant proportion of patients with wake-up stroke might benefit from reperfusion treatment and be promising candidates for intravenous thrombolysis. Different imaging approaches have been suggested to select wake-up stroke patients for thrombolysis, including multiparametric CT and MRI. Approaches currently under investigation involve the identification of tissue at risk of infarction independent from time by penumbral imaging and the identification of patients within the approved time-window for thrombolysis by the concept of DWI-FLAIR-mismatch. Both approaches are currently tested in large randomized controlled trials. The results of these trials are expected to change clinical practice by making available effective and safe treatment for a large group of acute stroke patients currently excluded from specific acute treatment.

## Conflict of Interest Statement

Götz Thomalla is the coordinating investigator of WAKE-UP and receives funding from the European Union Seventh Framework Programme [FP7/2007–2013] under grant agreement no: 278276 (WAKE-UP). D. Leander Rimmele has no conflicts of interest.
